# The Verdict of Science Concerning the Effects of Alcohol on Man

**Published:** 1875-06-15

**Authors:** N. S. Davis


### MEDICAL EXAMINER.

A

Semi-Monthly Journal of Medical Sciences.

EDITED BY N. S. DAVIS, M.D., AND F. H. DAVIS, M.D.

No. XII. CHICAGO, JUNE 15, 1875. VOL. XVI.

### Original Communications.

### THE VERDICT OF SCIENCE CONCERNING THE EFFECTS OF ALCOHOL ON MAN.

BY N. S. DAVIS, M.D.

*Read to the National Temperance Convention, June 2d, 1875.*

ALCOHOLIC liquids, as derived from the fermentation of various fruits and vegetable substances, have been known and used from an early period in the history of our race. Being derived from the grape or fruit of the vine chiefly, the name vinum, or wine, was naturally applied to all these liquids, until some time in the seventh century, when a liquid obtained from the fermentation of corn began to be called beer by the Saxons.

During the prevalence of the Alchemists' or Arabian school of chemistry, in the eleventh century, the vinous liquids in use began to be subjected to distillation, by which the active intoxicating constituent was obtained in a concentrated form, to which was applied the name "spirit of wine," and afterwards the word "alcohol." This last word appears to have been first used by the Arabians to designate an impalpable cosmetic powder used by the women of that day. It was afterwards applied to various subtle powders, and finally to spirit of wine. The first really scientific use of the term "alcohol" with which we are acquainted was by Lemert in his chemistry, published in 1698. For a long period after the discovery of spirit of wine or alcohol, it was used only as a solvent or menstruum in the preparation and preservation of other substances, while the fermented liquids continued to be used as drinks. The impure and diluted alcohols derived from distillation of fermented liquids, known as brandy, gin, rum, and whisky, are of modern origin, having been introduced into use within the last two or three centuries. Although we have a large variety of beverages derived from fermentation and distillation, known as wines, beers, and distilled spirits, yet ethylic, or absolute ether, universally known under the name of alcohol, constitutes the active, controlling ingredient in them all. The amount of this alcohol in the fermented drinks, called wines, beers, ales, etc., varies from four to twenty per cent.; while in the distilled spirits, called brandy, whisky, rum and gin, it constitutes from fifty to seventy-five per cent. Separate the alcohol from all these liquids, and the remainder would be capable of producing very little more effect on the human system than pure water. The juniper in gin, the hop in beer, and the vegetable acids and fecula in wines, are in quantities too small to exert any important influence, and hence may be omitted from our further consideration.

When we speak of alcohol, therefore, or of the effects of alcohol, throughout the remainder of this paper, we mean to include all alcoholic liquids, whether fermented or distilled. Until analytical and organic chemistry had made sufficient progress to show the composition of the more common articles of food and drink, no efforts were made to explain the special or physiological action of alcohol on the human system. All liquids containing it, were simply regarded as cordial or stimulant, and capable of supporting strength and life. When the chemico-physiological school of investigators, with Baron Liebig at its head, developed the fact that all alimentary substances were capable of being arranged into two classes, the nitrogenous and carbonaceous, they very naturally adopted the theoretical idea that the former when taken into the system were appropriated to the nourishment of the tissues, while the latter united with oxygen by a species of combustion, resulting in the development of animal heat and carbonic acid gas, and hence were familiarly styled "respiratory food."

Alcohol, being one of the purest of the carbonaceous class, and especially rich in carbon and hydrogen, was at once assigned a place at the head of the list of respiratory foods, and of supporters of animal heat. When taken into the living system, it was supposed to unite rapidly with the oxygen received through the lungs, evolving heat, and leaving as resultants carbonic acid gas and water; in this way its supposed heating and stimulating effects were explained.

The simplicity of the explanation, coupled with the high authority of Liebig, caused it to be almost universally accepted, although resting on a purely theoretical basis, without a single experimental fact for its support. It was not long, however, before Dr. Prout, of London, ascertained by direct experiment, that the presence of alcohol in the human system directly diminished the amount of carbonic acid gas exhaled from the lungs, and consequently there could be no combustion or oxydation of the alcohol by which it was converted into carbonic acid and water. Dr. Percy and others, by examination, found that alcohol taken in a dilute form into the stomach, was taken up without change of composition, and carried with the blood into all the organs and structures of the body; and that its presence could be easily detected by the proper chemical tests. The chemico-physiologists, however, still assuming that alcohol, being a hydro-carbon, must necessarily be used for maintaining temperature and respiration, suggested that the union of its elements with oxygen might be such as to result in forming acetic acid or aldehyde instead of carbonic acid gas. Hence they still sustained the popular belief that alcoholic drinks were capable of increasing both the temperature and strength of the human body. In the meantime, the process of experimentation went on. Dr. Boker, of Germany, by a well-devised and carefully-executed series of experiments, proved that the presence of alcohol in the living system, actually diminished the sum total of eliminations of effete matter daily; and consequently, that its presence must retard those molecular changes by which nutrition, secretion, and elimination are effected. In 1850, the writer of this paper prosecuted an extensive series of experiments to determine the effects of different articles of food and drink on the temperature of the body, and on the amount of carbonic acid excreted from the lungs. These experiments proved conclusively that, during the active period of digestion after taking any ordinary food, whether nitrogenous or carbonaceous, the temperature of the body is always increased; but after taking alcohol in the form of either fermented or distilled drinks, the temperature begins to fall within half an hour, and continues to decrease for from two to three hours. The extent and duration of the reduction of temperature was in direct proportion to the amount of alcohol taken. The results of this series of experiments were embodied in a paper read to the American Medical Association in May, 1851. [See *Northwestern Medical and Surgical Journal* for 1851.] A few years later, the experimental researches of Lallemand, Perrin and Duroy, proved conclusively that alcohol, when taken into the stomach, was not only absorbed and carried with the blood into all the organs and tissues of the body, but also that it was eliminated as alcohol, unchanged chemically, from the lungs, skin, and kidneys. The experiments of Prout were repeated, and his results confirmed by Sandras and Bouchardet, of France; W. A. Hammond, myself, and others of this country. Those of Boker were carefully repeated and varied by Anstie, of England, and Hammond, of this country. My own in reference to the effects of alcohol on animal heat have been repeated, and the results confirmed by a large number of observers, among whom are Drs. Richardson, Anstie, and Hammond. Those of Lallemand, in reference to the elimination of alcohol, have been equally confirmed, except the claim that the amount eliminated is not equal to the whole quantity taken. Hence the following propositions may be stated as fully established scientific facts:

*First.*—That alcohol, when taken diluted in the form of fermented or distilled spirits, is rapidly absorbed without change, carried into the blood, and with that fluid brought in contact with every structure and part of the human body.

*Second.*—That, while circulating in the blood, its presence retards those molecular or atomic changes by which nutrition, disintegration, and secretion are maintained, and the phenomena of life continued.

*Third.*—That its presence retards the elimination of waste matter, impairs nerve sensibility, lessens muscular excitability, and lowers the temperature of the body.

*Fourth.*—That a part, at least, of the amount taken is finally eliminated or thrown out of the system with the excretions, without having undergone any appreciable chemical change.

These facts are as well established as any in the domain of physiology, or in the whole field of natural science, and they point with all the clearness and force of a mathematical demonstration to the conclusion, that alcohol is in no sense food; neither furnishing material for the tissues, nor fuel for combustion, nor yet generating either nervous or muscular force. Having thus determined, experimentally, that alcohol is neither food nor a generator of force in the living body, the question recurs, What are its positive effects when taken in the ordinary manner? I answer, simply those of an anæsthetic and organic sedative. Like ether and chloroform, its presence diminishes the sensibility of the nervous system and brain, thereby rendering the individual less conscious of all outward and exterior impressions. This diminution of sensibility, or anæsthesia, is developed in direct ratio to the quantity of alcohol taken, and may be seen in all stages from simple exemption from all feeling of fatigue, pain, and idea of weight, exhibited by ease, buoyancy, hilarity, etc., to that of complete unconsciousness, and loss of muscular power. It is this anæsthetic effect of alcohol that has led to all the popular errors and contradictory uses, which have proved so destructive to human health and happiness. It has long been one of the noted paradoxes of human action, that the same individual would resort to the same alcoholic drink to warm him in winter, protect him from the heat in summer, to strengthen when weak or weary, and to soothe and cheer when afflicted in body or mind. With the facts now before us, the explanation of all this is apparent. The alcohol does not relieve the individual from cold by increasing his temperature; nor from heat by cooling him; nor from weakness and exhaustion by nourishing his tissues; nor yet from affliction by increasing nerve power; but simply by diminishing the sensibility of his nerve structure, and thereby lessening his consciousness of impressions, whether from cold, or heat, or weariness, or pain. In other words, the presence of the alcohol has not in any degree lessened the effects of the evils to which he is exposed, but has diminished his consciousness of their existence, and thereby impaired his judgment concerning the degree of their action upon him.

It is this property of alcohol to produce that sense of ease, buoyancy, and exhilaration, arising from a moderate diminution of nerve sensibility, that gives it the fascinating and delusive power over the human race, which it has wielded so ruinously for centuries gone by. But while the presence of alcohol diminishes the sensibility of the nervous structure, it also retards all the molecular changes, thereby diminishing the activity of nutrition, secretion, elimination, and the evolution of heat, constituting a true organic sedative. When taken in small quantities, repeated daily, the individual usually slowly increases in weight, not from increased nutrition, but from retarding the waste and retaining the old atoms longer in the tissues. By some investigators, this power to retard atomic changes and consequently to retain the old atoms, has been regarded as equivalent to nutrition, or the actual assimilation and addition of new atoms. It is on this basis that Dr. Hammond and a few others persist in representing alcohol as indirect food. The fallacy of such claim, and its mischievous tendency, will be fully apparent by reference to one of the plainest laws governing living animal matter. The law is, that all the phenomena of animal life are associated with and dependent on atomic changes, and that each individual cell or aggregation of bioplasm constituting an organic atom, has its determinate period of growth, maturity, and dissolution. Hence, to introduce into the living system any agent that will retard atomic change, is equivalent to retarding the phenomena of life. And if by retarding the atomic changes, cells or atoms are retained in the tissues longer than the natural duration of their activity, such retention may increase the bulk and weight, but in the same ratio it embarrasses the tissues with the presence of material which is constantly becoming inert and tending to degeneration. Consequently, the individual who thus increases his bulk and weight by taking just enough of the weaker alcoholic drinks daily to retard the processes of secretion and waste, in the same proportion diminishes his activity, his power of endurance, and his ability to resist the effects of morbid agents of every kind. This is abundantly illustrated by the thousands of beer and wine drinkers, who from twenty to twenty-five years of age, were muscular, active, capable of any reasonable endurance, with a weight of 150 pounds, but who, after moderately retarding atomic changes and retaining old atoms by the daily use of wine or beer, have acquired a weight of 200 pounds or more, and have lost their muscular activity and endurance to such an extent that an active exercise of twenty minutes would make them puff like a "heavy horse." It is this sedative effect of alcohol on the organic or molecular changes in the tissues, retaining waste and effete matter that ought to have been promptly disintegrated and thrown out, which impairs the vital properties, and predisposes or prepares the system to yield to morbid influences of any kind to which it may be exposed. And especially does this sedative effect of alcohol on the organic changes, when maintained by a moderate and continued use of the article, favor those degenerative changes, which result in tubercular, caseous, and fatty deposits in the lungs, liver, kidneys, heart, and arteries of the brain, and in materially shortening the duration of life. It is the same interference with the processes of nutrition and waste, only exerted more actively, that causes gastritis and delirium tremens in the excessive drinker of distilled spirits. If you ask for the special modus operandi of alcohol, how it produces its anæsthetic and sedative effect when taken into the human system, I answer, chiefly by its strong affinity for water and albumen. The two last named substances exist in the blood and all the tissues of the body, and for them alcohol has a strong chemical affinity. Hence, when it is present in the blood, it attracts the water from the blood corpuscles, causing them to become more or less corrugated, and inclined to adhere to one another as described by Dr. Richardson, of London, and diminishing the capacity of the blood to absorb oxygen or other gases from the air in the lungs ; and by its strong affinity for the albumen of the tissues, it retards the play of vital affinity between that substance and the other materials with which it is in contact, thereby retarding the molecular changes as already described. The paralyzing effect exerted on the vaso-motor as well as cerebro-spinal nervous structures by which sensibility is impaired, is owing partly to the direct anæsthetic properties of the alcohol, and partly to the diminished interchange of oxygen for carbonic acid gas in the process of respiration. That a part of the alcohol should be retained for a considerable length of time in the system by the affinities just mentioned, is very probable. Hence the late Dr. Anstie may have been correct in claiming that it was not all eliminated from the system within any limited period of time, and yet its retention would afford no proof that it was either appropriated as food or for the generation of force.

On the contrary, the catalytic influence of its presence retards both. If we scan the whole domain of physiology and pathology in connection with the logical deductions from the experimental researches by parties widely separated by time, space, nationality, and language, we shall be forced to the conclusion that alcohol as found in any or all of the fermented and distilled drinks, is neither stimulating, strengthening, nor nourishing to the human system, but simply anæsthetic and sedative. Consequently, it cannot be used in health without injurious effects proportioned to the quantity used and the frequency of its repetition. Its applicability as a remedy in the treatment of disease is extremely limited; so much so that it might be wholly dispensed with, without any injury to the sick, every intelligent physician being able to supply its place with other remedies of equal, if not greater, value in the limited number of cases in which it is applicable. Such we regard as the just and legitimate verdict of true science, regarding the effects of alcoholic drinks on man. We might amplify this paper by the citation of additional authorities and illustrative facts, until it would fill a volume ; but we have thought it more profitable and better fitting the present occasion, to limit it to a concise and plain statement of the present state of knowledge on this important subject.

---

DR. WILLIAM H. MIXON records (*American Practitioner*) a case in which, having no other instrument at hand, he tapped the bladder above the pubes with a bistoury. The patient made a good recovery.

DR. GEORGE H. BIXBY, of Boston, Mass., respectfully solicits from those members of the profession who have performed ovariotomy, their experience in regard to pregnancy occurring after that operation.

